# Commentary: Closing the gender gap in depression through the lived experience of young women – a response to ‘Don't mind the gap: Why do we not care about the gender gap in mental health?’, Patalay and Demkowicz (2023)

**DOI:** 10.1111/camh.12649

**Published:** 2023-03-20

**Authors:** Lucienne Spencer, Matthew Broome

**Affiliations:** ^1^ Institute of Mental Health University of Birmingham Birmingham UK; ^2^ Birmingham Women’s and Children’s NHS Foundation Trust Birmingham UK

**Keywords:** Depression, phenomenology, young women, gender gap, gender bias

## Abstract

Most mental health research largely ignores or minimises gender and age differences in depression. In ‘Don't mind the gap: Why do we not care about the gender gap in mental health?’, Patalay and Demkowicz identify a dearth of research on the causal factors of depression in young women. They attribute this to an over‐reliance on biological accounts of gender differences in depression. Patalay and Demkowicz conclude that a person‐centred approach that meaningfully engages with the reports of young women with depression is more likely to expose the social drivers of depression that impact this group. This commentary focuses on Patalay and Demkowicz's call to examine the patient's lived experience. We argue that there is an urgent need to reflect upon the methodologies involved in examining lived experience and how they can be best utilised. Ultimately, we advocate for an approach known as ‘phenomenological psychopathology’, through a phenomenological investigation of depression in young women, we can go some way towards closing the gender gap.

**Key Practitioner Message**

- Most mental health research largely ignores or minimises gender and age differences in depression.
- We risk falling back on a gender and age‐neutral account of depression, thus obstructing psychiatric knowledge and undermining the validity of the diagnostic criteria.
- Patalay and Demkowicz attempt to address this research gap by appealing to the ‘lived experience’ of young people with depression.
- We caution against a general methodology of ‘lived experience’ and instead advocate for phenomenological psychopathology.

## Summary

This article calls for a gendered understanding of depression in youth mental health. Patalay and Demkowicz observe an under‐researched gender difference in the way young women experience depression, which has not been taken seriously in the literature to date. According to Patalay and Demkowicz, the literature assumes a biological basis for this gender difference, citing factors such as hormones and neurotransmitters. The authors attribute a lack of interest in understanding gender differences to a false assumption that research in this area has been exhausted. Instead, Patalay and Demkowicz propose that we ought to turn to a social account of depression to better understand gendered risk factors. Gendered risk factors include vulnerability to sexual harassment and consequent victim blaming. Rather than attempting to alter the emotional response of women to entrenched misogynistic structures through ‘emotional regulation training’, we ought to make structural social changes.

In contrast to the biological approach, the authors argue that psychiatrists ought to examine the lived experience of depression. Patalay and Demkowicz propose that researchers ask participants about (a) their gendered experiences, (b) risk factors and (c) why they might be more vulnerable to depression. They conclude that a person‐centred approach that draws on the reports of young women is more likely to expose the hidden gender differences in depression. This commentary will focus on Patalay and Demkowicz's call to examine the lived experience of young women with depression.

## Funding and research bias

There is an urgent need to better understand the mental health of young women. According to a recent NHS digital survey, as children approach adulthood, rates of diagnosed psychiatric illness increase^1^: 15% of 7–10 year‐olds, 20.5% of 11–16 year‐olds, and 22% of 17–24 year‐olds have a diagnosable disorder (Newlove‐Delgado et al., 2022). This surge of poor mental health is particularly marked by emotional disorders such as depression, which are especially prevalent in women. Suicide has steadily increased in rates for females aged 24 and under (Suicides in England and Wales: 2021 registrations, ONS, 2022), and progressively more young people use self‐harm to manage stress and anxiety, with 42% of young women (17–24) having self‐harmed (Suicides in England and Wales: 2017 registrations, ONS, 2018). Nevertheless, little research focuses on understanding the causes of depression in young women.

What prevents us from developing a distinct account of depression in young people? Patalay and Demkowicz attribute this to a lack of funding in this area of research. This lack of funding is driven by an overreliance on biological models, a misconception that there is already research being funded in this domain and a general attitude of indifference towards this vital work. Patalay and Demkowicz also raise concerns regarding the power of funding bodies to dictate what issues are worth researching. To realign funding priorities, we identify a pressing need for greater inclusion in research funding bodies. As argued by Odedina and Stern (2021), a diverse funding body is more likely to recognise the significance of science and medical research that address the needs of marginalised groups. In mental health research, we suggest that funding bias can be ameliorated through greater representation in funding bodies, including the representation of those with lived experiences of psychiatric illness. Without the diversification of funding bodies, these research gaps will likely persist indefinitely.

Moreover, we observe that research gaps emerge through biases in the research methodology itself. Current research in mental health fails to pay sufficient attention to intersecting identities, such as being young or identifying as a woman. For instance, through ‘sampling bias’: a lack of inclusion of women or young people in research studies results in a one‐sided understanding of the given condition. A further form of bias is ‘data analytic bias’, where there is an equal sample of both men and women, or a wide age range including young people, yet the data is not analysed according to gender or age, and thus fails to illuminate intersectional experiences. As such, we have a skewed understanding of depression that only represents the experiences of dominantly positioned social groups.^2^

Given the dearth of research in this area, Patalay and Demkowicz call for an examination of the gender and age‐specific social structures that drive depression in young women. Their proposed methodology draws upon the first‐person reports of young women with depression.

## Lived experience

In the mental health literature, there is growing interest in examining the lived experience of psychiatric illness, particularly in youth mental health (Prebeg et al., 2022). Such work has done much to champion the valuable insight young people can offer into their own mental health. Yet, we caution against a generalised view of ‘lived experience’ as a methodology.

Patalay and Demkowicz argue that by examining the lived experience of young women with depression, we can identify and target the driving causal factors. The authors cite a study whereby young women are asked about the causes of depression in their demographic, and they provide valuable responses. However, it is important not to overextend the boundaries of lived experience. While some young women with depression may be able to offer insightful reflections on the vulnerability of young women to depression or, what the risk factors for depression might be in their demographic, the researcher will be fortunate if they encounter a participant whose expertise is in these areas. The expertise of the young female participant lies in their *own* lived experience of (a) being a woman and (b) having depression (plus any other intersectional vulnerabilities). Drawing out the narrative of their lived experience (be it through self‐narrative, pre‐structured interviews, or other methods) is an invaluable resource for gaining insight *into the nature of depression* in young women. Indeed, after examining a vast array of lived experiences of young women with depression, certain core social factors may emerge, e.g., most young women developed depression following sexual harassment. However, the expertise of the research participant lies in their lived experience.

We propose that it is worth reflecting on the following question: how do young women experience depression? Not what drives such depression, but how does it *feel*? As most mental health research largely ignores or minimises the role of gender differences, we risk perpetuating an understanding of depression that obscures the young female experience. By developing an account of how young women experience depression, we can improve diagnosis, treatment and our understanding of depression as a whole. We also avoid propagating hermeneutical injustices against young women that exclude their perspective from the interpretive framework of depression.^3^ While calls for ‘lived experience’ are growing in popularity in public mental health research, few provide concrete methodology. We argue that the best methodology for examining the lived experience of young women with depression can be found in the field of phenomenological psychopathology.

Phenomenological psychopathology has its roots in Karl Jaspers' seminal work *General Psychopathology* (1913/1963). Phenomenological psychopathology calls for an examination of the subjective phenomena of psychiatric illness (the lived experience). Advocates of the phenomenological method recognise that the first‐person illness narrative is central to our understanding of psychiatric illness. Phenomenological psychopathology has already done much to examine the lived experience of depression (Binswanger, 1960; Fuchs, 2013; Ratcliffe, 2015; Tatossian, 1975; Wilde, 2022). Various core phenomena of depression have been brought to the surface, such as an altered experience of time (Binswanger, 1960) and a breakdown of one's feeling of possibilities (Tatossian, 1975). However, what has received little attention is how young women experience depression. Thus, the essential core phenomena experienced by young women with depression are obscured from our current understanding. Through the Wellcome Trust funded project ‘Renewing Phenomenological Psychopathology,’ we propose to draw upon interdisciplinary and intersectional methodologies to address these gaps in the field.^4^ By examining the lived experience of specific marginalised groups, we can draw out distinct features of depression at these intersections. Through phenomenological psychopathology, we have the tools to develop a rich account of the experience of depression for young women, thus challenging the gender gap in mental health research.

## Conclusion

We agree that greater attention to the lived experience of young women with depression might shed light on the causal factors of the condition for this specific group. Gender‐neutral and age‐neutral accounts of depression suppress an understanding of the depression experienced by young women. While evaluating the lived experience of young women with depression may make the socially driven causal factors more visible, it is important to reflect upon the methodologies involved and how they can be best utilised. To overcome the gender gap in our understanding of depression, we must apply an intersectional approach that develops a distinct phenomenological account of depression in young women. We seek to achieve this through our Wellcome Trust funded project ‘Renewing Phenomenological Psychopathology’.

## Ethical information

No ethical approval was required for this commentary.

## Endnotes

^1^
We acknowledge that the term ‘illness’ and ‘disorder’ can be problematic, as it suggests that all forms of psychiatric ‘difference’ are necessarily pathological. We recognise the limits of this terminology.

^2^
One argument Patalay and Demkowicz launch against a biological understanding of gender difference is that it fails to show up in research across ‘all countries and contexts’ and thus must be socially driven. However, the research bias we discuss here may explain why gender difference does not appear universally.

^3^
For more on ‘hermeneutical injustice’, see Fricker (2007).

^4^
Further information can be found on the project website: https://www.birmingham.ac.uk/research/mental‐health/renewing‐phenomenological‐psychopathology/renewing‐phenomenological‐psychopathology.aspx.
